# A Case Report on the Use of Pharmacological Intervention in the Treatment of Diffuse Axonal Injury From Road Traffic Accidents

**DOI:** 10.25122/jml-2019-1012

**Published:** 2019

**Authors:** Ignacio Previgliano, Marcela A. Soto

**Affiliations:** Neurology Chair, Critical Care Course, Maimonides University, Buenos Aires, Argentina

**Keywords:** traumatic brain injury, cerebrolysin, diffuse axonal injury

## Abstract

We report a case of traumatic brain injury treated with Cerebrolysin, a neurorecovery stimulating agent. Our therapeutic approach was based on the pathophysiology of traumatic brain injury and, in particular, of diffuse axonal injury. The patient registered marked improvement in mood and cognitive performance, indicating the effectiveness of multimodal and multidisciplinary interventions after traumatic brain injury.

## Case Report

**Figure 1:**
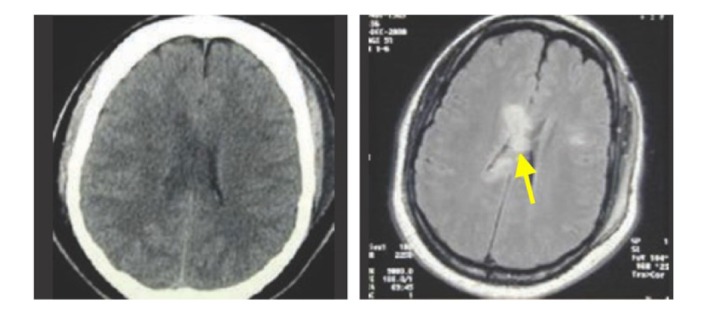
CT and MRI scans showing lesions compatible with diffuse axonal injury (DAI). The arrow signals a shear lesion at the corpus callosum.

A 24-year-old man suffered a car accident, arriving at the emergency room (ER) in a comatose state with a Glasgow Coma Scale (GCS) of 4/15, miotic pupils, decerebrate posture when painful stimuli were applied and spontaneous hyperventilation. A CT scan showed changes compatible with diffuse axonal injury, confirmed further by MRI (Figure 1).

The patient was transferred to the intensive care unit (ICU). An intraparenchymal fiberoptic device was placed in the left parietal lobe for intracranial pressure (ICP) monitoring. He spent 45 days in the ICU, during which he developed several medical complications, including nosocomial pneumonia, septic shock, and ventilator dependency.

He was transferred to a rehabilitation facility in a patent vegetative state with nocturnal mechanical ventilation requirements. He has never experienced epileptic seizures, had no symptoms of infections, and his laboratory values were normal.

We decided to administer Cerebrolysin 30 ml/day for ten days. The Argentinian regulatory office had recently approved the drug, and we had no prior experience of its use in traumatic brain injury (TBI), so we decided to adopt the dosage recommended in the literature [1-3].

Our therapeutic approach was based on the pathophysiology of traumatic brain injury and, in particular, of the diffuse axonal injury. Diffuse axonal injury (DAI) had been traditionally considered an acceleration-deceleration injury resulting in widespread neuronal damage: small petechial hemorrhages in the corpus callosum and dorsolateral pons quadrants and axonal disruption (clusters and Wallerian degeneration) associated with brain edema, which could be seen on CT and MRI scans [4].

Nevertheless, recent evidence has shown that DAI is present with any type of brain injury, focal or otherwise, especially in those patients dying immediately after TBI as well as those who remained in a persistent vegetative state or severely disabled [5,6].

The cytoskeletal damage of DAI was initially assumed to occur rapidly, due to the transmission of shear forces throughout the brain. Modern research shows that tearing of the axons occurs rarely; a progressive disruption of the axonal membrane is the norm, not only in severe but also in moderate and mild TBI cases. Mitochondrial disruption could play a central role in this process [7]. Very recent evidence has pointed out that axonal damage and degeneration are not always associated with neuronal death, as it was assumed up to now. It may be possible for neurons to survive such insults and even attempt to regenerate, providing a further chance of recovery [8]. This means neuroplasticity and neurotrophic action, which was indicative of the Cerebrolysin administration [9].

**Figure 2:**
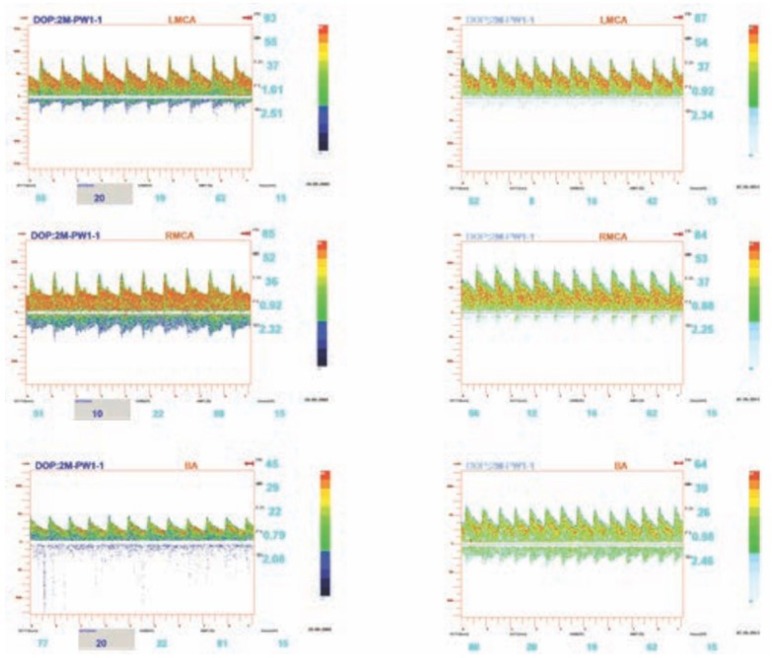
Blood flow velocities after and before Cerebrolysin administration showed an improvement in diastolic velocities and calculated CPP, rising from 48 mmHg to 61 mmHg.

A transcranial Doppler (TCD) was performed in order to establish cerebral blood flow velocities in the Circle of Willis and to calculate cerebral perfusion pressure (CPP) using Belfort's formula (Figure 2) [10].

By the 7^th^ day of Cerebrolysin administration, the patient began to respond to simple orders and could be weaned from nocturnal mechanical ventilation.

A new TCD was performed, which showed a significant improvement in blood flow velocities and CPP as compared to baseline (Figure 2).

On the 45^th^ Cerebrolysin administration, the patient was able to perform a MiniMental State Examination, scoring 18 points out of 30 He also presented pyramidal signs of the four extremities with a vicious position in flexion of both upper arms.

We decided to perform another Cerebrolysin treatment cycle with the 10 ml/day dosage for 21 days, the dose recommended for cognitive disorders, three months after the first administration [11]. We also decided to administer botulinum toxin in the upper extremities and the vocal cords. After the second Cerebrolysin treatment cycle, the patient improved the MMSE score to 25/30, and a full cognitive battery was performed, including Benton's test and verbal fluency (Table 1). He also improved his motor abilities, gained weight and muscular mass. Occupational therapy was reinforced.

**Table 1:** Cognitive evaluation using Mini-Mental State Examination, Benton and Verbal Fluency tests.

	After Cycle 1	After Cycle 2	After Cycle 3
**MMSE**	18	25	28
**Benton (general efficiency)**	NA	4	9
**Verbal Fluency (mean/normal)**	NA	7.5/18	13.5/18

A third Cerebrolysin treatment cycle was scheduled after another three months using the same dosage. After that, the patient improved his cognitive abilities, resulting in an MMSE of 28/30 (faults in recalling) and improvement in Benton's test and verbal fluency (Table 1).

A fourth Cerebrolysin cycle was performed with improvement in mood and attention, meanwhile memory and verbal fluency did not change.

## Conclusion

The patient showed unexpected improvement following the treatment. Considering the encouraging results and taking into consideration the overall safety profile of Cerebrolysin, we started administering it as routine treatment in difusse axonal injury due to traumatic brain injury.
